# Combined Administration of (*R*)-Ketamine and the mGlu2/3 Receptor Antagonist LY341495 Induces Rapid and Sustained Effects in the CUMS Model of Depression via a TrkB/BDNF-Dependent Mechanism

**DOI:** 10.3390/ph15020125

**Published:** 2022-01-21

**Authors:** Anna Rafało-Ulińska, Piotr Brański, Agnieszka Pałucha-Poniewiera

**Affiliations:** Department of Neurobiology, Maj Institute of Pharmacology, Polish Academy of Sciences, Smętna Street 12, 31-343 Kraków, Poland; anna.rafalo@gmail.com (A.R.-U.); nfbransk@cyf-kr.edu.pl (P.B.)

**Keywords:** antidepressant, BDNF, CUMS, LY341495, (*R*)-ketamine, (*S*)-ketamine

## Abstract

Ketamine is an effective, rapid-acting antidepressant drug (RAAD), but it induces side effects. To overcome these challenges, attempts have been made to use safer enantiomer ((*R*)-ketamine) or mGlu2/3 receptor antagonists, which induce ketamine-like effects and enhance its action. Here, we propose combining these two strategies to investigate the antidepressant-like effects of low doses of two ketamine enantiomers in combination with a low dose of the mGlu2/3 receptor antagonist LY341495. Rapid and sustained antidepressant-like effects were assessed in C57BL/6J mice using the tail suspension test (TST) and the chronic unpredictable mild stress (CUMS) model of depression in stress-naïve mice. ELISA was used to measure BDNF levels. In the TST, low doses of both (*S*)-ketamine and (*R*)-ketamine were potentiated by a subeffective dose of LY341495. However, in the CUMS model, only (*R*)-ketamine was able to induce long-lasting anti-apathetic and anti-anhedonic effects when coadministered with low-dose LY341495. The mechanism of this drug combination was dependent on BDNF and AMPA receptor activity. ELISA results suggest that the hippocampus might be the site of this action. MGlu2/3 receptor antagonists, in combination with (*R*)-ketamine, may serve as potential RAADs, with a high efficiency and low risk of side effects.

## 1. Introduction

Ketamine, a new rapid-acting antidepressant drug (RAAD) that induces fast and long-lasting therapeutic action [1] and has been introduced for treatment of resistant major depressive disorder [2], also produces serious side effects that can be dangerous, especially in psychiatric patients (e.g., psychostimulant effects, dissociation, short-term memory impairments and abuse potential) [3,4]. Therefore, research is still being conducted to reduce these effects. Several studies show that the use of (*R*)-ketamine instead of (*S*)-ketamine, which is currently an approved psychiatric drug [2], might have beneficial therapeutic effects while reducing side effects, especially those related to psychostimulatory action [5,6,7]. Attempts have also been made to lower the therapeutic dose of ketamine by coadministering it with substances that have a convergent mechanism of action. Animal studies have shown that mGlu receptor ligands, especially mGlu2/3 receptor antagonists, may meet these requirements. These compounds not only induce rapid antidepressant-like effects in animal models of depression but also share many mechanisms of antidepressant activity with ketamine, including involvement of the BDNF and mTOR pathways and dependence on AMPA receptor activation and serotonergic system activity [8,9,10,11]. Furthermore, an important role of the mGlu2 receptor in the mechanism of the antidepressant action of ketamine in mice was found by Zanos et al. [12], who showed that the antidepressant-like effects of ketamine and its metabolite, (*2R,6R*)-HNK, were absent in Grm−/− mice and were blocked by the mGlu2/3 receptor agonist LY379268.

Our previous studies have shown that the therapeutic dose of ketamine could be lowered by coadministration with an mGlu2/3 receptor antagonist. We found that LY341495 not only potentiated subeffective doses of ketamine in screening tests that assess the antidepressant effects of drugs (e.g., the forced swim test, FST) in rats both 40 min and 24 h after administration [13,14] but also produced a rapid antidepressant-like effect in a depression model based on chronic unpredictable mild stress in mice when coadministered with a subeffective dose of ketamine [15]. Zanos et al. [12] recently confirmed the synergistic antidepressant effect of ketamine and LY341495 in the FST in CD-1 mice, both 1 h and 24 h after administration, and also demonstrated a similar effect with the ketamine metabolite (*2R,6R*)-HNK coadministered with LY341495. Moreover, subliminal doses of (*2R,6R*)-HNK and LY341495 produced a synergistic enhancement of the cortical gamma power of QEEG [12].

Ketamine is a racemic mixture of (*R*)-ketamine and (*S*)-ketamine, and the mechanisms of the pharmacological actions of each enantiomer differ. These differences include various affinities for the NMDA receptor complex [16], different effects on the regulation of monoamine release in various brain structures [17], involvement in various molecular mechanisms [18] and different therapeutic potentials, such as (*R*)-ketamine exhibiting a longer-lasting and stronger antidepressant activity than (*S*)-ketamine in animal models of depression [5,6,7].

However, it is not known what role each enantiomer plays in the mechanism of the mGlu2/3 receptor antagonist potentiating the antidepressant effects of ketamine. Determining the role that (*R*)- and (*S*)-ketamine play in inducing antidepressant effects when coadministered with LY341495 may be of significant importance for practical applications in the treatment of depression, given the different action mechanisms of both ketamine enantiomers and, consequently, the various side effects that may accompany their application. Therefore, we decided to investigate the effect of the mGlu2/3 receptor antagonist LY341495 on the antidepressant action of (*R*)-ketamine and (*S*)-ketamine in mice. First, a screening test (the TST) was used to investigate the potential antidepressant action of the tested compounds after a single injection [19]. Then, the CUMS model of depression in mice was used to investigate how rapid and sustained the effects of the tested drug combinations are [20]. In addition, a series of experiments was planned to investigate the mechanisms involved in the potentiation of ketamine enantiomers by LY341495.

While investigating the mechanisms, we focused on the possible role of BDNF, its receptor (tropomyosin receptor kinase B (TrkB)) and the AMPA receptor in the observed effects, based on studies that have identified these factors as necessary for the antidepressant effects of ketamine and mGlu2/3 receptor antagonists [8,10,11,21,22,23]. For this purpose, a series of behavioral studies and the measurement of BDNF levels in several mouse tissues were planned.

## 2. Results

### 2.1. Rapid Antidepressant-like Effects of (S)-Ketamine and (R)-Ketamine Coadministered with LY341495 in the TST

**Figure 1:**
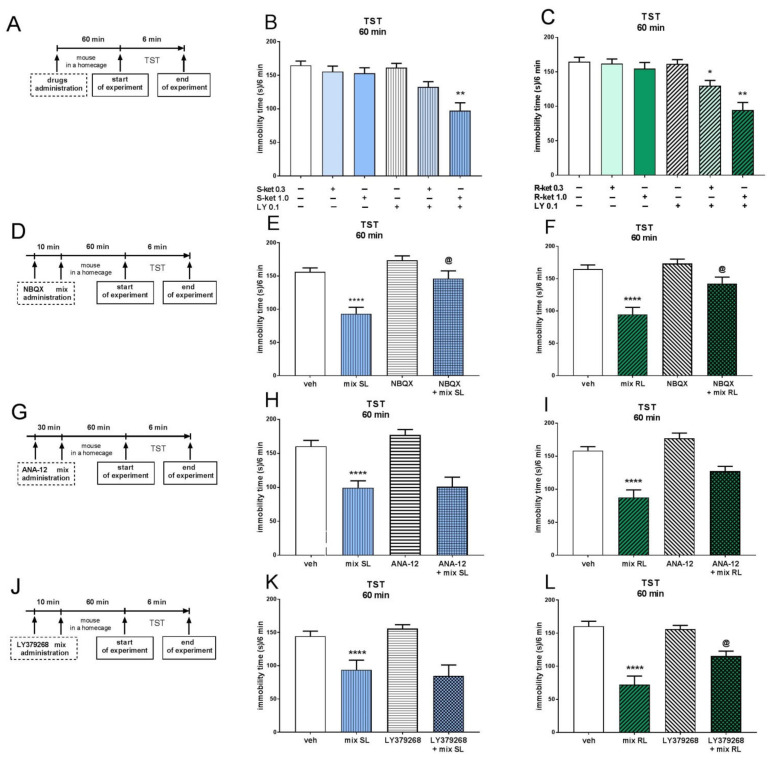
The antidepressant-like effects of (*S*)-ketamine (blue panel) and (*R*)-ketamine (green panel) coadministered with LY341495 in the TST. (**A**) schedule of experiments B and C; (**B**) the antidepressant-like effects of (*S*)-ketamine coadministered with LY341495; (**C**) the antidepressant-like effects of (*R*)-ketamine coadministered with LY341495; (**D**) schedule of experiments E and F; (**E**) influence of NBQX on the antidepressant-like effect of the mix SL; (**F**) influence of NBQX on the antidepressant-like effect of the mix RL; (**G**) schedule of experiments H and I; (**H**) influence of ANA-12 on the antidepressant-like effect of the mix SL; (**I**) influence of ANA-12 on the antidepressant-like effect of the mix RL; (**J**) schedule of experiments K and L; (**K**) influence of LY379268 on the antidepressant-like effect of the mix SL; (**L**) influence of LY379268 on the antidepressant-like effect of the mix RL. The values are expressed as the means ± SEM and were analyzed by two-way ANOVA. * *p* < 0.05, ** *p* < 0.01 drug × drug interaction; **** *p* < 0.0001 mix SL or mix RL main effect; ^@^ *p* < 0.05 drug × drug interaction (N = 8–9). Mix SL—(*S*)-ketamine 1 mg/kg + LY 341495 0.1 mg/kg; mix RL—(*R*)-ketamine 1 mg/kg + LY 341495 0.1 mg/kg.

Two-way ANOVA revealed that (*S*)-ketamine (1 mg/kg) induced a significant antidepressant-like effect in the TST when coadministered with a subeffective dose of LY341495 (0.1 mg/kg) 60 min before the TST [interaction: F(1,28) = 9.592, *p* < 0.01]. A lower dose of (*S*)-ketamine (0.3 mg/kg) coadministered with LY341495 (0.1 mg/kg) did not induce a significant effect in this test [interaction: F(1,28) = 1.796, *p* > 0.05] (Figure 1B). An interaction was found between each tested dose of (*R*)-ketamine (1 mg/kg and 0.3 mg/kg) and LY341495 (0.1 mg/kg) when coadministered 60 min before the TST [interactions: F(1,27) = 11.43, *p* < 0.01 and F(1,28) = 4.55, *p* < 0.05, respectively] (Figure 1C).

Investigation of the mechanism of potentiation of the antidepressant-like effect of each ketamine enantiomer by LY341495 in the TST showed that these effects were blocked by AMPA receptor antagonist NBQX (10 mg/kg), which was administered 10 min before the mixture of (*S*)-ketamine (1 mg/kg) and LY341495 (0.1 mg/kg) (mix SL; Figure 1E) or the mixture of (*R*)-ketamine (1 mg/kg) and LY341495 (0.1 mg/kg) (mix RL; Figure 1F) [interactions: F(1,27) = 4.265, *p* < 0.05; F(1,28) = 4.963, *p* < 0.05, respectively]. On the other hand, pretreatment with the TrkB receptor antagonist ANA-12 (0.5 mg/kg) had no effect on the antidepressant-like action of mix SL [F(1,26) = 0.503, *p* > 0.05] (Figure 1H) or mix RL [F(1.26) = 1.598; *p* > 0.05] (Figure 1I). Two-way ANOVA also showed that administering the mGlu2/3 receptor agonist LY379268 (3 mg/kg) 10 min before mix SL did not influence the immobility time of the mice during the TST [interaction: F(1,30) = 0.793, *p* > 0.05] (Figure 1K). However, the antidepressant-like effect of mix RL was significantly reduced by pretreatment with LY379268 (3 mg/kg) [interaction: F(1,32) = 7.12, *p* < 0.05] (Figure 1L).

### 2.2. The Effects of (S)-Ketamine and (R)-Ketamine in the CUMS Model of Depression

**Figure 2:**
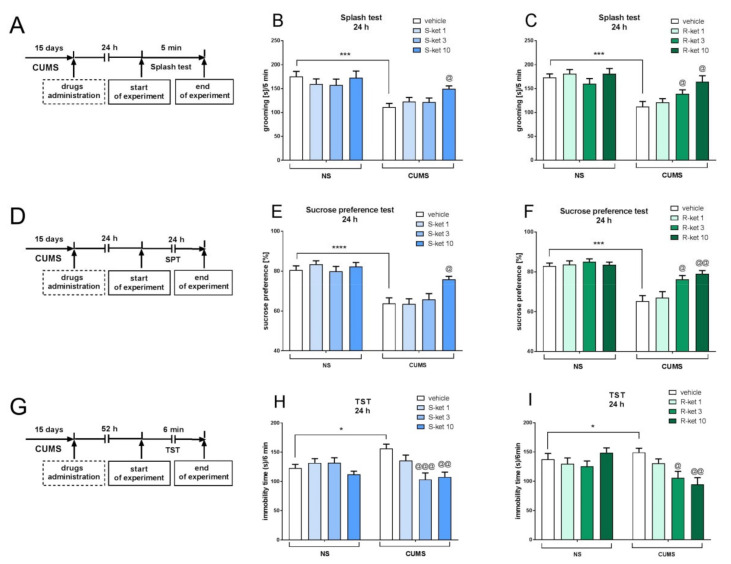
The antidepressant-like effects of (*S*)-ketamine (blue panel) and (*R*)-ketamine (green panel) in the CUMS model of depression. (**A**) schedule of experiments B and C; (**B**) (*S*)-ketamine effect in the splash test; (**C**) (*R*)-ketamine effect in the splash test; (**D**) schedule of experiments E and F; (**E**) (*S*)-ketamine effect in the sucrose preference test; (**F**) (*R*)-ketamine effect in the sucrose preference test; (**G**) schedule of experiments H and I; (**H**) (*S*)-ketamine effect in the TST; (**I**) (*R*)-ketamine effect in the TST. The values are expressed as the means ± SEM and were analyzed by two-way ANOVA. ^@^ *p* < 0.05, ^@@^ *p* < 0.01, ^@@@^ *p* < 0.001 CUMS × drug interaction; * *p* < 0.05, *** *p* < 0.001, **** *p* < 0.0001 (CUMS effect, *t*-test) (N = 10–12).

To determine the active and subactive doses of (*S*)- and (*R*)-ketamine in the CUMS model of depression, each enantiomer was tested at three doses (1, 3 and 10 mg/kg) in the splash test, the SPT and the TST. In the splash test, two-way ANOVA revealed that the CUMS-induced effect [F(1,43) = 18, *p* < 0.001] was significantly reversed by (*S*)-ketamine (10 mg/kg) [CUMS × drug interaction: F(1,43) = 6.927, *p* < 0.05] (Figure 2B). (*R*)-ketamine also increased grooming in CUMS mice during the splash test, and two-way ANOVA showed the main effect of CUMS on the behavior of mice [F(1,42) = 17.32, *p* < 0.001], as well as the interactions between CUMS and (*R*)-ketamine 3 mg/kg [F(1,42) = 7.015, *p* < 0.05] and (*R*)-ketamine 10 mg/kg [F(1,42) = 6.453, *p* < 0.05] (Figure 2C). Similar results were found in the SPT, where the CUMS effect [F(1,36) = 29.02, *p* < 0.0001] was significantly reversed by (*S*)-ketamine administered at a dose of 10 mg/kg [CUMS × drug interaction: F(1,36) = 5.832, *p* < 0.05] (Figure 2E). (*R*)-ketamine was able to reverse the CUMS effect [F(1,36) = 33.82, *p* < 0.001] not only at a dose of 10 mg/kg [CUMS × drug interaction: F(1,36) = 11.6, *p* < 0.01] but also at a dose of 3 mg/kg [CUMS × drug interaction: F(1,36) = 4.739, *p* < 0.05] (Figure 2F). A dose-dependent antidepressant-like effect of both ketamine enantiomers in CUMS mice was also found in the TST. Two-way ANOVA revealed the main effect of CUMS in this test [F(1,43) = 4.342, *p* < 0.05], and (*S*)-ketamine was found to reverse the CUMS effect at doses of 10 mg/kg [CUMS × drug interaction: F(1,43) = 7.294, *p* < 0.01] and 3 mg/kg [CUMS × drug interaction: F(1,44) = 12.81, *p* < 0.001] (Figure 2H). Similarly, (*R*)-ketamine reversed the CUMS-induced effect [F(1,41) = 4.971, *p* < 0.05] at doses of 10 mg/kg [CUMS × drug interaction: F(1,41) = 12.17, *p* < 0.01] and 3 mg/kg [CUMS × drug interaction: F(1,40) = 5.107, *p* < 0.05] (Figure 2I). Importantly, neither (*S*)-ketamine nor (*R*)-ketamine induced any effect in any of the tests performed on NS mice at any tested dose. Based on the results, a dose of 1 mg/kg was chosen as a subactive dose for both ketamine enantiomers in the CUMS model of depression.

### 2.3. The Effects of (S)-Ketamine and (R)-Ketamine Coadministered with LY341495 in the CUMS Model of Depression

**Figure 3:**
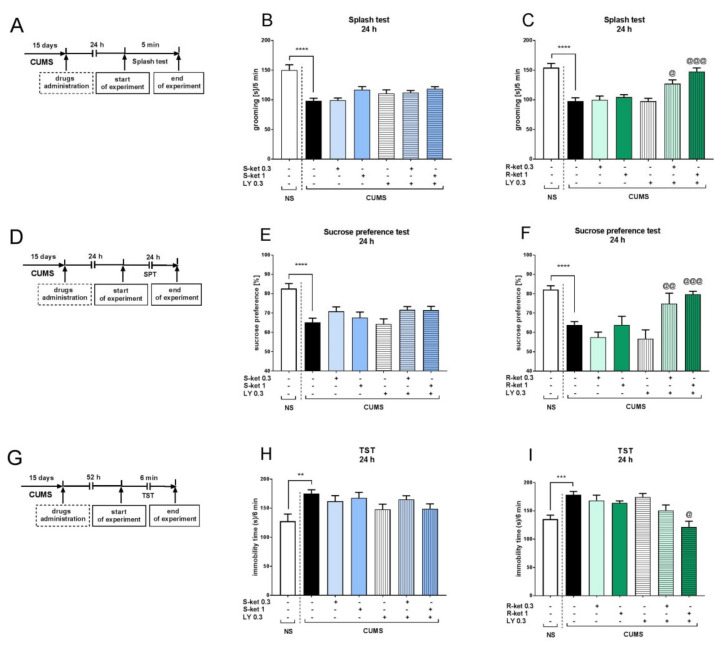
The effects of (*S*)-ketamine (blue panel) and (*R*)-ketamine (green panel) coadministered with LY341495 in the CUMS model of depression. (**A**) schedule of experiments B and C; (**B**) effects of (*S*)-ketamine coadministered with LY341495 in the splash test; (**C**) effects of (*R*)-ketamine coadministered with LY341495 in the splash test; (**D**) schedule of experiments E and F; (**E**) effects of (*S*)-ketamine coadministered with LY341495 in the sucrose preference test; (**F**) effects of (*R*)-ketamine coadministered with LY341495 in the sucrose preference test; (**G**) schedule of experiments H and I; (**H**) effects of (*S*)-ketamine coadministered with LY341495 in the TST; (**I**) effects of (*R*)-ketamine coadministered with LY341495 in the TST. The values are expressed as the means ± SEM and were analyzed by two-way ANOVA. ^@^ *p* < 0.05, ^@@^ *p* < 0.01, ^@@@^ *p* < 0.001 drug × drug interaction; ** *p* < 0.01, *** *p* < 0.001, **** *p* < 0.0001 (CUMS effect, *t*-test) (N = 10–12).

As none of the ketamine enantiomers induced any effects in the NS group, all further studies in the CUMS model were performed on the stressed group. The NS group was restricted to the animals administered the vehicle to determine whether a given behavioral parameter changed under chronic stress. The splash-test *t* test revealed that CUMS mice had a considerably shorter grooming time than NS mice (*p* < 0.0001) (Figure 3B). Two-way ANOVA showed that a subeffective dose of (*S*)-ketamine (1 mg/kg) coadministered with a subeffective dose of LY341495 (0.3 mg/kg) did not induce any changes in the behavior of CUMS mice [interaction: F(1,30) = 1.294, *p* > 0.05]. A lack of interaction between the drugs was also found for the lower tested doses of (*S*)-ketamine (0.3 mg/kg) and LY341495 (0.3 mg/kg) [F(1.30) = 0.001, *p* > 0.05] (Figure 3B). However, a subeffective dose of (*R*)-ketamine (1 mg/kg) coadministered with a subeffective dose of LY341495 (0.3 mg/kg) induced a significant anti-apathetic effects during the splash test [drug × drug interaction: F(1.28) = 17.21, *p* < 0.001]. Furthermore, a lower dose of (*R*)-ketamine (0.3 mg/kg) also induced a significant effect in this test when coadministered with LY341495 [drug × drug interaction: F(1.29) = 5.351, *p* < 0.05] (Figure 3C). In the SPT, CUMS mice consumed less sucrose than non-stressed controls (*t*-test, *p* < 0.0001; Figure 3E). Neither of the tested doses of (*S*)-ketamine (1 or 0.3 mg/kg) combined with a subeffective dose of LY341495 (0.3 mg/kg) produced an effect in this test [drug × drug interactions: F(1,32) = 0.934, *p* > 0.05 and F(1,32) = 0.128, *p* > 0.05, respectively] (Figure 3E). On the other hand, (*R*)-ketamine (1 or 0.3 mg/kg) coadministered with LY341495 (0.3 mg/kg) significantly reversed CUMS-induced anhedonia in the SPT [drug × drug interactions: F(1,32) = 13.41, *p* < 0.001; F(1,30) = 10.35, *p* < 0.01, respectively] (Figure 3F). Similar effects were observed in the TST, with CUMS mice having longer immobility times than NS mice (*t*-test, *p* < 0.01; Figure 3H). (*S*)-ketamine (1 or 0.3 mg/kg) coadministered with LY341495 (0.3 mg/kg) did not change the behavior of the mice during this test [drug × drug interactions: F(1,28) = 0.225, *p* > 0.05; F(1,30) = 3.319, *p* > 0.05, respectively] (Figure 3H), while a 1 mg/kg dose of (*R*)-ketamine coadministered with LY341495 (0.3 mg/kg) induced a significant antidepressant-like effect in CUMS mice [drug × drug interaction: F(1,31) = 6.92, *p* < 0.05] (Figure 3I).

### 2.4. Sustained Effects of (R)-Ketamine Coadministered with LY341495 in the CUMS Model of Depression

**Figure 4:**
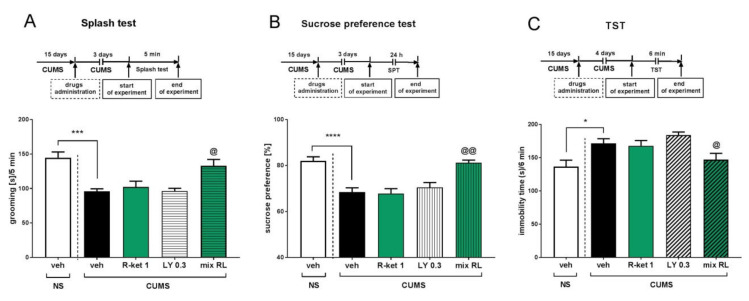
Sustained effects of (*R*)-ketamine coadministered with LY341495 in the CUMS model of depression. (**A**) schedule of experiment and effects in the splash test; (**B**) schedule of experiment and effects in the sucrose preference test; (**C**) schedule of experiment and effects in the TST. The values are expressed as the means ± SEM and were analyzed by two-way ANOVA. ^@^ *p* < 0.05, ^@@^ *p* < 0.01 drug × drug interaction; * *p* < 0.05, *** *p* < 0.001, **** *p* < 0.0001 (CUMS effect, t-test) (N = 10–12).

The sustained effects of (*R*)-ketamine (1 mg/kg) and LY341495 (0.3 mg/kg) (mix RL) were studied in the CUMS model of depression 3 days after their coadministration. In the splash test, a two-way ANOVA revealed the anti-apathetic effect of mix RL [drug × drug interaction: F(1,27) = 4.298, *p* < 0.05] (Figure 4A). The anti-anhedonic effect of mix RL 3 days after treatment was also observed in the SPT [drug × drug interaction: F(1,35) = 9.336, *p* < 0.01] (Figure 4B). Moreover, in the TST, a small but significant interaction between (*R*)-ketamine and LY341495 was found by two-way ANOVA [F(1,28) = 4.826, *p* < 0.05] (Figure 4C).

### 2.5. Mechanisms of the Effects of (R)-Ketamine Coadministered with LY341495 in the CUMS Model of Depression

**Figure 5:**
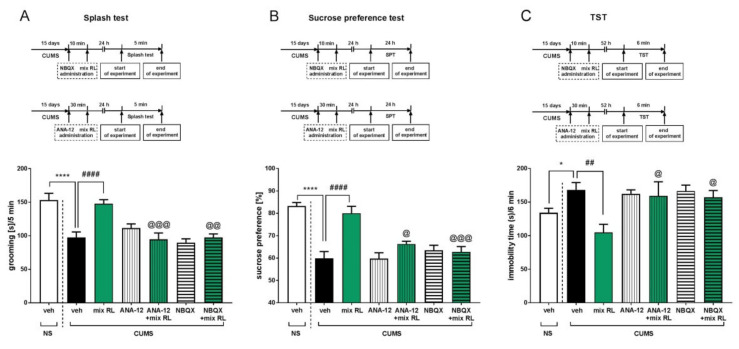
Mechanisms of the effects of (R)-ketamine coadministered with LY341495 in the CUMS model of depression. (**A**) schedule of experiments and effects of NBQX or ANA-12 pretreatment on the antidepressant-like effects of the mix RL in the splash test; (**B**) schedule of experiments and effects of NBQX or ANA-12 pretreatment on the antidepressant-like effects of the mix RL in the sucrose preference test; (**C**) schedule of experiments and effects of NBQX or ANA-12 pretreatment on the antidepressant-like effects of the mix RL in the TST. The values are expressed as the means ± SEM and were analyzed by two-way ANOVA. ^@^ *p* < 0.05, ^@@^ *p* < 0.01, ^@@@^ *p* < 0.001 drug × drug interaction; ## *p* < 0.01, #### *p* < 0.0001 mix RL main effect; * *p* < 0.05, **** *p* < 0.0001 (CUMS effect, *t* test) (N = 10–12). Mix RL–(*R*)-ketamine 1 mg/kg + LY 341495 0.3 mg/kg.

To investigate the possible mechanism of the enhancement of a subeffective dose of (*R*)-ketamine (1 mg/kg) by a subeffective dose of LY341495 (0.3 mg/kg), antagonists of the TrkB receptor and the AMPA receptor (ANA-12 and NBQX, respectively) were administered prior to administration of mix RL. In the splash test, CUMS mice had shorter grooming times than NS mice (*t*-test, *p* < 0.0001) (Figure 5A). Two-way ANOVA performed within the CUMS group showed that ANA-12 (0.5 mg/kg) given 30 min before mix RL reversed the anti-apathetic action of mix RL [ANA-12 × mix RL interaction: F(1,28) = 19.8, *p* < 0.001]. NBQX (10 mg/kg) administered 10 min before mix RL also prevented its action in the splash test [NBQX × mix RL interaction: F(1,28) = 11.11, *p* < 0.01] (Figure 5A). In the SPT, the previously observed effect of CUMS on sucrose preference was confirmed (*t*-test, *p* < 0.0001). A two-way ANOVA showed that ANA-12 (0.5 mg/kg) significantly reduced the anti-anhedonic effect of mix RL [ANA-12 × mix RL interaction: F(1,27) = 6.386, *p* < 0.05]. Moreover, the AMPA receptor antagonist NBQX (10 mg/kg) completely reversed the activity of mix RL during the SPT [NBQX × mixed RL interaction: F(1,27) = 14.37, *p* < 0.001] (Figure 5B). Finally, in the TST, CUMS mice were found to have longer immobility times than NS mice (*t*-test, *p* < 0.05; Figure 5C), and a two-way ANOVA showed the main effect of CUMS [F(1,27) = 11.85, *p* < 0.01], as well as the interactions between mix RL and ANA-12 (0.5 mg/kg) [F(1,27) = 4.774, *p* < 0.05] and mix RL and NBQX (10 mg/kg) [F(1,27) = 6.643, *p* < 0.05] (Figure 5C).

### 2.6. The Effects of (S)-Ketamine and (R)-Ketamine Coadministered with LY341495 on the Locomotor Activity of Mice

**Figure 6:**
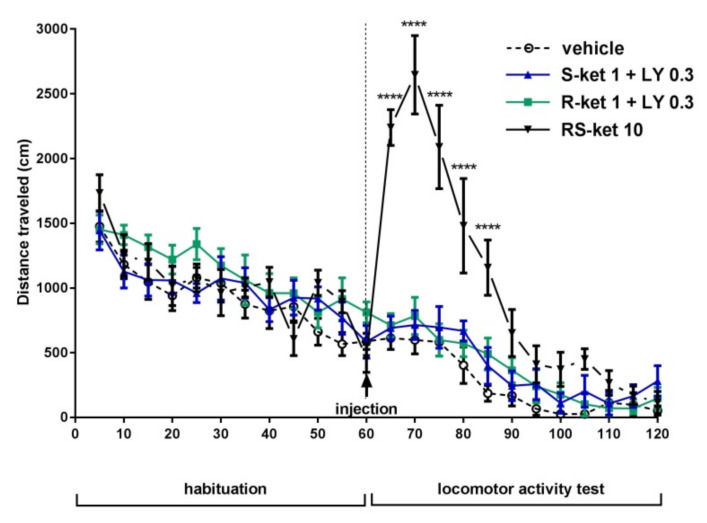
The effects of (*S*)-ketamine and (*R*)-ketamine coadministered with LY341495 on the locomotor activity of mice. The values are expressed as the means ± SEM and were analyzed by repeated-measures ANOVA. **** *p* < 0.0001 vs. vehicle (N = 8).

A two-way repeated-measures ANOVA showed that the reference drug, (RS)-ketamine, administered at a dose of 10 mg/kg induced a rapid increase in locomotor activity ([F (1,7) = 12, *p* < 0.01) (Figure 6). Bonferroni’s multiple comparisons test showed a significant effect of (RS)-ketamine (10 mg/kg) at all time points tested, ranging from 5 to 25 min after injection (*p* < 0.0001). The effect of (RS)-ketamine-induced hyperactivity reached a peak 10 min after injection and then gradually decreased (Figure 6). (*R*)-ketamine (1 mg/kg) coadministered with LY341495 (0.3 mg/kg) did not change the locomotor activity of the mice [F(1,7) = 5.451; *p* > 0.05] (Figure 6). Furthermore, when compared with control mice, (*S*)-ketamine (1 mg/kg) coadministered with LY341495 (0.3 mg/kg) did not induce hyperlocomotion [F(1,7) = 2.179, *p* > 0.05] (Figure 6).

### 2.7. The Effects of (R)-Ketamine Coadministered with LY341495 on BDNF Levels in the CUMS Model of Depression

**Figure 7:**
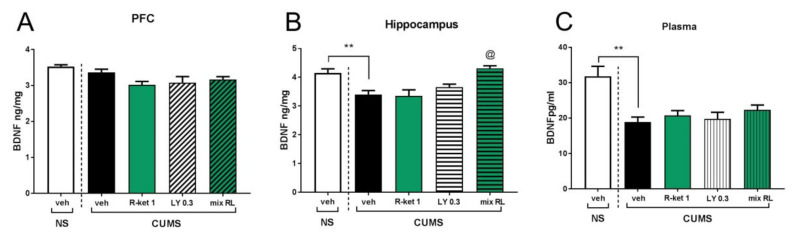
The effects of (*R*)-ketamine coadministered with LY341495 on BDNF levels in the CUMS model of depression. (**A**) effects in the PFC; (**B**) effects in the hippocampus; (**C**) effects in the plasma. The values are expressed as the means ± SEM and were analyzed by two-way ANOVA. ^@^ *p* < 0.05 drug × drug interaction; ** *p* < 0.01 (CUMS effect, *t* test) (N = 8).

The PFC *t* test revealed that compared with the NS group, CUMS did not influence the BDNF level (*p* > 0.05), and a two-way ANOVA showed that (R)-ketamine (1 mg/kg) given jointly with LY341495 (0.3 mg/kg) did not change BDNF levels in CUMS mice in this brain region [interaction: F(1,26) = 2.45, *p* > 0.05] (Figure 7A). On the other hand, in the hippocampus, CUMS mice were found to have lower BDNF levels than NS control mice (*t*-test, *p* < 0.01). Moreover, a two-way ANOVA revealed that (R)-ketamine (1 mg/kg) coadministered with LY341495 (0.3 mg/kg) significantly reversed the CUMS-induced effect on this brain structure [drug × drug interaction: F(1,25) = 5.56, *p* < 0.05] (Figure 7B). Plasma BDNF levels in the CUMS group were significantly lower than those in the NS control group (*t*-test, *p* < 0.01). However, this low BDNF level was not changed by any applied drug used alone or in combination with another drug [drug × drug interaction: F(1,26) = 0.039, *p* > 0.05] (Figure 7C).

## 3. Discussion

The results of the present study indicate that the antidepressant-like effects of (*R*)-ketamine, but not (*S*)-ketamine, in C57BL/6J mice may be potentiated by the mGlu2/3 receptor antagonist LY341495 in the CUMS model of depression. However, in the antidepressant drug-screening test in naïve mice (TST), both ketamine enantiomers were enhanced by LY341495. Each of these effects was studied separately because the results of the screening test and the depression model may indicate that the tested compounds have different antidepressant (AD) actions. The screening test (in this case, the TST) shows the potential effect of classic ADs in mice shortly after a single administration, although these substances are known to produce therapeutic effects in patients after several weeks of systematic administration [24]. Thus, the TST results obtained for naïve mice 60 min after a single administration of a drug or a combination of drugs indicate the possibility of an antidepressant effect but do not indicate the speed at which the antidepressant effect could appear in patients. On the other hand, the study of antidepressant effects in the CUMS model of depression allows us to predict whether this effect will be fast and permanent. This is because classic ADs require several weeks of administration to counteract CUMS-induced behavioral effects, while RAADs work after a single or short-term administration [15,20,25,26].

In the TST, we found that both subeffective doses of (*S*)-ketamine and (*R*)-ketamine were potentiated by a subeffective dose of LY341495, although (*S*)-ketamine required a higher dose than (*R*)-ketamine to induce this effect. Sixty minutes after the administration of each drug combination, the locomotor activity of the animals did not differ from that of the control group, which excludes a possible false-positive effect of the drug on the mobility of mice and can thus be interpreted as an antidepressant-like effect. Overall, the TST results indicate that the antidepressant-like effects of both ketamine enantiomers were able to be potentiated by the mGlu2/3 antagonist. These results are in agreement with the results obtained by Zanos et al. [12], which showed that coadministration of subeffective doses of (*RS*)-ketamine or the (*R*)-ketamine metabolite (*2R,6R*)-HNK with LY341495 reduced immobility time in the FST in CD1 mice 1 and 24 h after administration.

The mechanisms of (*R*)-ketamine and (*S*)-ketamine potentiation by LY341495 appear to be similar, as the antidepressant-like effects of both ketamine enantiomers coadministered with LY341495 were blocked by the AMPA receptor antagonist but were not altered by the TrkB receptor antagonist, suggesting that the effects were not dependent on BDNF activity but were related to AMPA receptor activation. These results generally confirm previous data on the mechanisms of the antidepressant-like effects of ketamine and mGlu2/3 antagonists in the TST and the FST when tested separately at similar times after administration [10,12]. However, the lack of ANA-12 efficacy might be related to the properties of this drug, which was detected in the mouse brain 30 min after intraperitoneal injection but was only able to inhibit TrkB receptors homogeneously throughout the brain after 4 h [27]. It is worth noting that both ketamine and LY341495 are known to cause rapid BDNF release and that ketamine action was dependent on BDNF activity 30 min after its administration [11,21,23].

However, an important difference in the antidepressant-like effects of (*S*)-ketamine and (*R*)-ketamine coadministered with LY341495 was found when pretreating with the mGlu2/3 receptor agonist LY379268. It was observed that the mGlu2/3 receptor agonist did not influence the antidepressant effect of (*S*)-ketamine coadministered with LY341495, while it significantly reduced the antidepressant effect of (*R*)-ketamine coadministered with LY341495, indicating that in the latter case, the mechanism of action of the tested substances was dependent on the activation of mGlu2/3 receptors. These results are consistent with a study by Zanos et al. [12], which showed that both ketamine and (*2R,6R*)-HNK had no antidepressant-like effect in mice when mGlu2/3 receptors were activated by LY379268. However, our research directly shows a key role of the mGlu2/3 receptor in the mechanism of rapid action of (*R*)-ketamine but not (*S*)-ketamine, especially since when (*S*)-ketamine was coadministered with an mGlu2/3 antagonist, the behavioral effect of this combination was not changed in the presence of an agonist of these receptors. This may indicate that in the drug combinations we used, (*R*)-ketamine was a key element, the mechanism of action of which depended on the mGlu2/3 receptor.

To assess how fast and sustained the antidepressant effects of the tested combinations of ketamine enantiomers with LY341495 were, we used CUMS, a recognized and well-validated model of depression based on chronic, mild environmental stress [20,28]. In this model, we analyzed three parameters that reflect the core symptoms of depression: reduced grooming time in the splash test, which reflects apathy [29]; decreased sucrose preference, which indicates anhedonia [30]; and increased immobility time in the TST, which relates to helplessness [24].

The research began by determining the dose-effect curves for (*R*)-ketamine and (*S*)-ketamine, as neither the literature data nor our own research indicated the subliminal doses in the CUMS model. Our previous studies using the splash test and the sucrose preference test showed that both (*S*)-ketamine and (*R*)-ketamine produced antidepressant effects at a dose of 10 mg/kg in the CUMS model 24 h after dosing [6]. Therefore, we chose to test 10 mg/kg, as well as lower doses of 3 and 1 mg/kg, of both ketamine enantiomers. Since the 3 mg/kg dose changed some mouse behaviors in the CUMS model, the 1 mg/kg dose was chosen as the subeffective dose and was combined with a subeffective dose of LY341495 (0.3 mg/kg), which was selected based on our previous experiments performed using a similar experimental design [15]. It is worth noting that none of the ketamine enantiomers induced an antidepressant-like effect in non-stressed animals, which confirms several previous findings that in stress-naïve mice, ketamine and its enantiomers have no effect 24 h after administration [6,15,31,32].

We demonstrated that a subeffective dose of (*R*)-ketamine coadministered with a subeffective dose of LY341495 abolished the CUMS-induced behavioral effects in all tests. Additionally, in the splash test and the sucrose preference test, a statistically significant effect was demonstrated for the lower dose of (*R*)-ketamine (0.3 mg/kg) coadministered with LY341495. On the other hand, the use of subeffective doses of (*S*)-ketamine coadministered with a subeffective dose of LY341495 did not change any behavioral parameters in this model of depression, which indicates that only one ketamine enantiomer, (*R*)-ketamine, participates in the mechanism of interaction with the mGlu2/3 receptor antagonist, rapidly and effectively reducing the effects of chronic stress in a mouse model of depression. Moreover, this effect persisted 3 days after coadministration of subeffective doses of (*R*)-ketamine and LY341495, which was observed in all behavioral parameters analyzed. Therefore, it can be assumed that (*R*)-ketamine plays a key role in the mechanism of the enhancement of the antidepressant effect of ketamine by LY341495 in CUMS, as shown in a previous study [15]. The studied combination of drugs appears to be not only therapeutically effective but also safe, as in the hyperlocomotion test, no effect of (*R*)-ketamine coadministered with LY341495 on animal behavior was observed. In contrast, (*RS*)-ketamine induced strong hyperlocomotion 5–25 min after administration of a 10 mg/kg dose, which is typically used as a therapeutic dose in animal studies, indicating a potential psychostimulatory effect.

In further research, we decided to focus on a possible mechanism responsible for the effective interaction of these two substances. Using behavioral tests, we investigated the role of the AMPA receptor and TrkB receptor using their antagonists (NBQX and ANA-12, respectively). In all the analyzed behavioral tests in the CUMS model, we found that the antidepressant effect of the combination of (*R*)-ketamine and LY341495 depended on the activation of the AMPA receptor, indicating that these effects are neuronal-activity-dependent. This is similar to the mechanism observed in the screening test performed 60 min after the compounds were administered. These results are consistent with numerous studies by other authors using various behavioral techniques that clearly indicate that the rapid and sustained antidepressant-like effects of ketamine, its enantiomers and some metabolites are blocked by an AMPA receptor antagonist [6,7,33,34].

The TrkB receptor antagonist also completely abolished the antidepressant effect of (*R*)-ketamine coadministered with LY341495 in the CUMS model in the splash test, the sucrose preference test and the TST. In this case, however, the result differed from that obtained in the TST 60 min after administration, where the TrkB antagonist had no effect on the antidepressant effect of the tested compounds. This clearly shows that the mechanism of the antidepressant-like action of (*R*)-ketamine coadministered with LY341495 in the screening test differed from that observed 24 h after administration in the CUMS model, where dependence on BDNF activation has been demonstrated. Sun et al. [26] showed that the sustained antidepressant effect of ketamine in the CUMS model in rats was blocked by ANA-12. Our experiment indicates that this effect is most likely related to the action of (*R*)-ketamine. This result is in agreement with previous observations that the mechanisms underlying the various pharmacological effects of (*R*)-ketamine, but not (*S*)-ketamine, are dependent on BDNF activation [35,36], including antidepressant-like activity [6,21,37]. Importantly, LY341495, similar to ketamine, rapidly increased BDNF release in primary neuronal cultures, and this effect was blocked by incubation with an AMPA receptor antagonist [11]. A similar effect was not observed with classic ADs, such as fluoxetine or desipramine [11], indicating that rapid, activity-dependent BDNF release might be a common mechanism of RAAD action, such as the drug combination used in this study.

Based on CUMS results, we investigated BDNF levels in brain structures potentially associated with antidepressant activity (PFC and hippocampus), as well as in the plasma of mice previously subjected to the CUMS procedure [22]. In the PFC, we did not observe any differences in BDNF levels in non-stressed and CUMS mice, and furthermore, no changes in BDNF levels were induced by any of the drugs tested. In contrast, CUMS mice were found to have significantly lower BDNF levels in the hippocampus than control mice, and the combination of (*R*)-ketamine and LY341495 reversed these effects, which may indicate a relationship between BDNF levels in the hippocampus and therapeutic effects.

Our findings support one of the leading hypotheses on the mechanism of the rapid antidepressant effects of ketamine, which is that ketamine plays a key role in inducing a distinctive type of synaptic plasticity in the hippocampus [22]. In this hypothesis, ketamine is postulated to block NMDA receptors, which prevents calcium entry into the cell and blocks the phosphorylation of eukaryotic elongation Factor 2 (eEF2), eventually leading to a rapid increase in BDNF protein levels and a subsequent potentiation of AMPA receptor-mediated excitatory postsynaptic potentials in the hippocampus [21,22,38]. This AMPA receptor-mediated synaptic potentiation has been proposed to play a critical role in the rapid antidepressant effect of ketamine [21,22,38]. Interestingly, it has recently been shown that BDNF-TrkB signaling at synapses in the CA3-CA1 regions of the hippocampus is essential for the rapid antidepressant action of ketamine [39]. Therefore, it appears that this specific localization may be related to the antidepressant effect of the coadministration of (*R*)-ketamine and LY341495. Interestingly, it has been recently reported using the same ELISA technique that low-dose ketamine in combination with guanosine not only counteracted chronic corticosterone-induced antidepressant-like effects in mice but also reversed BDNF reduction in the mouse hippocampus, though not in the mouse PFC [40]. However, it should be remembered that several studies have reported that exposure to different types of chronic stress, including chronic social defeat stress (CSDS) and CUMS, decreased the expression of BDNF not only in the hippocampus but also in the PFC [7,8,26].

A significant decrease in the BDNF level of stressed mice was also demonstrated in the plasma, but none of the tested compounds, either administered separately or in combination, changed this level. Thus, it seems that the plasma BDNF level does not reflect the changes observed in the behavior of animals. This result appears to be consistent with previous observations in patients treated with ketamine, which show that the drug does not alter plasma BDNF levels in patients successfully treated with ketamine [41]. It also supports the conclusion of Arosio et al. [42], who stated in a recent review that blood BDNF levels cannot be used as a biomarker in clinical practice.

In summary, our data support the use of mGlu2 receptor antagonists in combination with (*R*)-ketamine as potential RAADs with a high efficiency and low risk of side effects in experimental therapeutic trials for the treatment of depression.

## 4. Materials and Methods

### 4.1. Animals and Housing

The experiments were performed on male C57BL/6J mice (Charles River, Germany). Animals were maintained under standard laboratory conditions in terms of temperature (22 ± 2 °C), humidity (55 ± 10%) and lighting (light phase 6:00-18:00), with free access to food and tap water. The mice used in the CUMS model of depression were 5 weeks old at the beginning of the experiment, while the mice used in the TST and the locomotor activity test were 8 weeks old at the beginning of the experiment, ensuring that the behavioral studies in all experimental groups were performed on animals of similar ages. Each experimental group in the behavioral studies consisted of eight to twelve animals. All experimental procedures were conducted in accordance with the guidelines of the National Institutes of Health Animal Care and Use Committee and were approved by the Second Local Ethics Committee in Kraków, Poland. The three Rs principles were applied in the planning and execution of the experiments. Every effort was made to reduce the number of animals used and to avoid and minimize animal suffering.

### 4.2. Compounds

(*S*)-(+)-Ketamine hydrochloride (Tocris Cookson, Ltd., Bristol, UK), (*R*)-ketamine hydrochloride (Cayman Chemicals, Ann Arbor, USA), LY341495 disodium salt (Tocris Cookson, Ltd.), NBQX disodium salt (Tocris Cookson Ltd.), LY379268 disodium salt (Tocris Cookson, Ltd.) and ketamine hydrochloride solution (Bioketan, Biowet, Puławy, Poland) were diluted with 0.9% NaCl. ANA-12 (Tocris Cookson Ltd.) was dissolved in 2% DMSO. Herein, 0.9% NaCl or 2% DMSO was used as a vehicle. All compounds and vehicles were injected intraperitoneally (i.p.) at a constant volume of 10 mL/kg.

### 4.3. Tail Suspension Test

The experiments were carried out in accordance with the procedure described by Steru et al. [19], which had been previously performed in our laboratory [43]. The mice were habituated to the testing room for 30 min before the experiments. Animals were attached by their tails to a table with adhesive tape (placed approximately 1 cm from the distal end of the tail). The total duration of immobility was assessed during a 6-min period. The mice were considered immobile when they hung down passively with no limb movements.

### 4.4. CUMS Procedure

**Figure 8:**
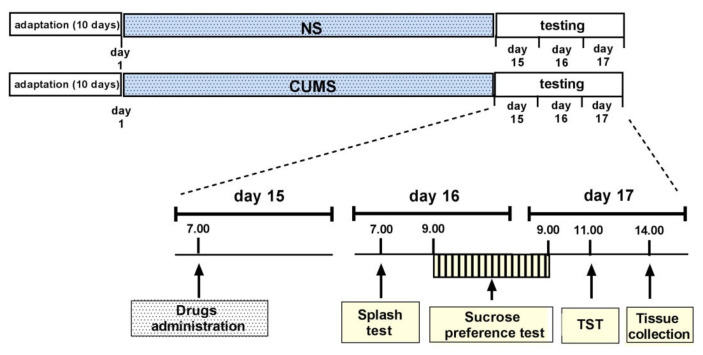
The schedule of the CUMS experiments.

The CUMS procedure was performed as previously described, with the necessary modifications [43]. The experimental scheme is presented in Figure 8. The animals were divided into two groups: control mice (unstressed, designated NS) and mice subjected to CUMS. NS mice and CUMS mice were kept in separate rooms. After 10 days of adaptation to room conditions, during which all animals were handled and weighed, the CUMS procedure was started. The following stressors were used: restraint stress (30–60 min), cage tilting (45°; 4–12 h), predator smell (rat) (15 min–4 h), wet bedding (3–6 h), removal of sawdust (2–4 h), placing a mouse in the cage of another mouse (1–2 h), reversed light-dark cycle (48 h), substitution of sawdust with 37 °C water (60–90 min), placing 3 individuals in an empty cage (30–60 min), placing 3 individuals in a cage with 37 °C water (60–90 min) and overcrowding (18 individuals; 60 min). Two or three stressors were used daily, depending on their duration. Some stressors were used at night (tilted cage, inverted day/night cycle). A break of at least two hours between stressors was used. The stressors began at different times of the day between 7:00 and 11:00 and were administered in random order; the duration of each stressor was changed to maintain the principle of unpredictability.

On the 15th day after the beginning of the CUMS procedure, the tested compounds ((*S*)-ketamine, (*R*)-ketamine and LY341495) were given separately or jointly at 7:00. NBQX (10 mg/kg) or ANA-12 (0.5 mg/kg) was administered 10 min and 30 min, respectively, before the mixtures of (*R*)- or (*S*)-ketamine and LY341495. Twenty-four hours after treatment, the splash test (see Section 4.5) was performed (starting at 7:00), followed by the sucrose preference test (SPT (see Section 4.6), starting at 9:00). Two hours after the end of the SPT, the TST was performed (see Section 4.3), and three hours later, the animals were sacrificed by rapid decapitation, trunk blood was collected and brain structures (PFC and hippocampus) were dissected and frozen at −80 °C. The same schedule of behavioral tests was used 72 h after drug administration. The control animals (NS) were tested with the same schedule as the CUMS animals.

### 4.5. Splash Test

The splash test was performed as previously described [15], with minor modifications. The test was performed under dimmed lighting in a room to which the animal had been adapted for 30 min. A high-viscosity 10% sucrose solution was sprayed on the dorsal coat of the mice to stimulate self-grooming behavior. The sprayer allowed the delivery of a fixed volume (approximately 0.2 mL) of sucrose solution, and each mouse received five sprays. Then, the duration of grooming was recorded for five minutes.

### 4.6. Sucrose Preference Test

The SPT was performed according to Strekalova and Steinbusch [44] and Pałucha-Poniewiera et al. [15]. The test was performed two times: one day before the CUMS procedure began, to determine the baseline value of sucrose preference, and 26 h (or 3 days) after drug administration to examine the effects of the test substances. Twenty-four hours before the first SPT, mice were allowed to consume a 2.5% sucrose solution for 2 h to diminish the effect of neophobia. Mice were given a free choice between a bottle with a 1% sucrose solution and another bottle with tap water for 24 h. The position of the bottles was switched after 12 h. No previous food or water deprivation was applied before the test. At the beginning and end of the test, the bottles were weighed, and the liquid consumption was calculated. The preference for sucrose was calculated as a percentage of consumed sucrose solution in terms of the total amount of liquid drunk.

### 4.7. Locomotor Activity

The locomotor activity of the mice was measured in Plexiglas locomotor activity chambers (40 × 20 × 15 cm) in a 20-station photobeam activity system (Opto-M3 Activity Meter, Columbus Instruments, Columbus, OH, USA), where the animals were individually placed for an acclimation period of 60 min. Then, they were administered an i.p. drug injection. Immediately after injection, each mouse was returned to the chamber. The total distance traveled during a 60-min experimental session was measured and stored every 5 min.

### 4.8. BDNF Levels

The total BDNF concentrations were measured with an ELISA kit purchased from R & D Systems, Inc. Minneapolis, MN, USA (Catalog Number SBNT00) according to the manufacturer’s recommendations. The total BDNF concentrations were determined by comparing the samples to the standard curve. All samples were thawed on ice and diluted (2-fold for plasma and 100-fold for tissue lysates in Calibrator Diluent RD5K). Fifty microliters of Assay Diluent RD1-123 was added, and 50 µL of each sample or standard was added to a 96-well microtiter plate in triplicate. The plate was incubated for 2 h at room temperature with vigorous mixing (550 rpm, ThermoMixer C, Eppendorf, Hamburg, Germany). Each well was rinsed four times with 400 µL of wash buffer. An enzyme-linked monoclonal antibody specific for BDNF was added (200 µL) to the wells and incubated at room temperature for 1 h with vigorous mixing (550 rpm, ThermoMixer C). Next, the wells were washed four times with 400 µL of wash buffer to remove any unbound antibody-enzyme reagent. Two hundred microliters of substrate solution was added and incubated in the dark for 1 h. Then, the plates were read at 450 nm and 570 nm using a spectrophotometer (Synergy HTX multimode reader machine; BioTek Instruments Inc., Winooski, VT, USA). The optical density was corrected by subtracting the readings at 570 nm from the readings at 450 nm. A standard curve was generated by a four-parameter logistic-curve fit. The data were linearized by plotting the log of the total BDNF concentrations versus the log of the O.D. The best-fit line was determined by regression analysis. Finally, the total BDNF concentration was normalized to the protein concentration of each sample. For standard curve fitting and sample-data interpolation, GraphPad Prism version 9.2.0 for Windows was used (GraphPad Software, San Diego, CA, USA).

### 4.9. Data Analysis

All the results obtained are expressed as the mean ± standard error of the mean (SEM). The statistical analyses of all behavioral data were performed using GraphPad Prism 7.00 (GraphPad Software, San Diego, CA, USA). Two-way ANOVA was used to analyze the interactions between CUMS and the drug, as well as the effects of drug combinations in the splash test, SPT and TST. Locomotor activity data were evaluated by repeated-measures ANOVA, followed by Bonferroni’s multiple comparisons test. The results obtained using the ELISA method were analyzed by two-way ANOVA using GraphPad Prism version 9.2.0 for Windows (GraphPad Software, San Diego, CA, USA). The results were considered significant if the *p* values were below 0.05.

## 5. Conclusions

The results of the current study show that combined subeffective doses of an mGlu2/3 receptor antagonist and (*R*)-ketamine synergistically exerted antidepressant-like effects in mice, both in a screening test and in the CUMS model of depression based on chronic environmental stress. Based on previous studies and the current results, we believe that this synergistic action may result from a common mechanism of drugs with mGlu2-receptor-antagonist activity and (*R*)-ketamine, which involves rapid BDNF release and is dependent on neuronal activity and AMPA receptor signaling [11]. This indicates that the mechanisms of action of both LY341495 and (*R*)-ketamine might be related to increased glutamate transmission and neuronal depolarization. This effect may occur via inhibition of GABA interneurons by (*R*)-ketamine and/or blockade of presynaptic Glu2/3 autoreceptors by LY341495 [11,12], which may finally lead to a synergistic effect.

## Data Availability

All relevant data is presented in the manuscript, raw data is available upon request from the corresponding author.
